# Active Video Gaming Intervention in Adults With Mobility Impairments: Concurrent Mixed Methods Single-Arm Pilot Study

**DOI:** 10.2196/73764

**Published:** 2026-08-26

**Authors:** Laurie A Malone, Christen J Mendonca, Sangeetha Mohanraj, Mohanraj Thirumalai

**Affiliations:** 1 Department of Occupational Therapy School of Health Professions University of Alabama at Birmingham Birmingham, AL United States; 2 School of Health Professions University of Alabama at Birmingham Birmingham, AL United States; 3 Division of General Internal Medicine and Population Sciences Heersink School of Medicine University of Alabama at Birmingham Birmingham, AL United States

**Keywords:** exergaming, physical disability, adapted physical activity, active video gaming, mixed methods

## Abstract

**Background:**

Adults with mobility impairments have limited opportunities for physical activity due to environmental barriers and lack of accessible exercise options. Active video games (AVGs) have shown promise for increasing physical activity by integrating movement into engaging gameplay. However, most commercial AVG controllers are not usable by those with severe mobility limitations. The GAIMplank is a novel adaptive game controller that translates trunk movements into gameplay, addressing this accessibility gap and enabling seated participation for individuals with limited lower-extremity function.

**Objective:**

This study aimed to assess feasibility and acceptability of a 6-week GAIMplank-based intervention in adults with mobility impairments and to evaluate participants’ perceived exertion, enjoyment, and engagement during gameplay. In addition, qualitative feedback was used to characterize participants’ experiences with the intervention and to provide insight into implementation considerations for adapted exergaming in this population.

**Methods:**

In total, 6 adults (2 female; mean age 65.2, SD 12.4 years) with mobility impairments due to stroke or Parkinson participated in a concurrent mixed methods single-arm pilot study. The intervention consisted of twice-weekly AVG sessions over 6 weeks using the GAIMplank controller to play video games. Self-report baseline measures included physical function, exercise expectations, and level of physical activity. During gameplay sessions, ratings of perceived exertion (RPEs), engagement, and enjoyment (0-10 rating scale) were collected. Additionally, physical activity and exergame enjoyment surveys were completed after sessions 1, 5, and 12. After the intervention, acceptability, appropriateness, and feasibility of the intervention (0-5 rating scale) were assessed. Qualitative data were collected through session comments and postintervention semistructured interviews. Descriptive statistics were used to summarize quantitative data, thematic analysis for qualitative data, and integration to examine convergence.

**Results:**

Participants reported moderate RPEs during gameplay (median RPE 5, IQR 3-6) and high levels of enjoyment and engagement (median scores of 9, IQR 6-10 and median 10, IQR 8-10, respectively). The intervention was well-received, with high scores on the acceptability, appropriateness, and feasibility measures. Qualitative findings highlighted engagement and enjoyment of the games, as well as participants’ perceptions of mental stimulation, sustained attention, and positive emotional experiences during gameplay. Several participants also described a “masking effect,” in which physical effort was not perceived as traditional exercise due to immersion in gameplay. Technical challenges related to controller responsiveness were reported, but they did not appear to substantially reduce overall engagement.

**Conclusions:**

Findings from this mixed methods pilot study suggest that AVGs, delivered using an adapted controller such as the GAIMplank, appear feasible and enjoyable in this small pilot sample, supporting the need for further evaluation in larger, controlled studies. While standardized cognitive or mental health outcomes were not assessed, participant feedback indicates potential psychological benefits that warrant further investigation. Future studies should explore the long-term effects of AVG interventions and identify strategies for maximizing their benefits.

**Trial Registration:**

ClinicalTrials.gov NCT05310279; https://clinicaltrials.gov/study/NCT05310279

## Introduction

Adults with disabilities experience persistent inequities in physical activity participation and are substantially less likely to meet aerobic and muscle-strengthening physical activity guidelines than adults without disabilities. National surveillance analyses in the United States indicate large gaps in the number of adults with disabilities meeting the guidelines, underscoring the need for scalable, accessible approaches that can be performed in safe and convenient settings [1]. In parallel, public health guidance, including the World Health Organization’s 2020 recommendations, explicitly recognizes that people living with disability should engage in regular physical activity, while also emphasizing the importance of adapting activities to ability and context [2]. For adults with mobility impairments, barriers such as transportation, limited program availability, inaccessible facilities, fatigue, and difficulty identifying enjoyable activities can constrain participation in conventional exercise options. Technology-enabled approaches have been highlighted as one pathway to reduce access barriers by enabling activity in controlled environments (eg, home or clinic) and supporting adaptation, personalization, and engagement [3].

Active video games (AVGs), also known as exergames, have emerged as a promising approach to promote physical activity across diverse populations, including older adults and individuals with disabilities [4-8]. Recent evidence indicates that AVGs can support improvements in physical health, functional ability, and participation when appropriately tailored to user needs [9-13]. For example, case-based and small-scale intervention studies have demonstrated the feasibility of integrating exergaming into rehabilitation and community settings, with reported benefits in mobility, engagement, and user satisfaction [10]. In addition to physical outcomes, AVGs have been shown to foster social interaction and enhance engagement through interactive and immersive gameplay [4-8,14]. By integrating physical movements within gameplay, AVGs offer an engaging alternative to traditional exercise modalities and may help reduce sedentary behavior, particularly for individuals who face barriers to conventional physical activity opportunities [15-17].

Exergaming embeds physical activity within a motivating task. Recent work highlights its feasibility and potential benefits when specifically tailored to user capabilities and settings [18,19]. Research indicates that AVGs can facilitate light-to-moderate intensity physical activity, contributing to improved physical fitness and reduced sedentary behavior [15,16,20]. Recent evidence further suggests that exergaming can meaningfully contribute to weekly physical activity and improve functional outcomes like balance and mobility, although results vary by game type, dose, and population [21,22]. In older adults, AVGs have been shown to enhance muscular strength, cardiorespiratory fitness, mobility, and balance [23-26]. Systematic review evidence also suggests that exergaming may promote positive emotional experiences across populations, with reported effects on outcomes such as happiness, anxiety, depressive symptoms, vitality, and intrinsic motivation [27]. More recent review evidence in older adults further suggests potential benefits for mood and social engagement, while a 2026 meta-analysis found a small but statistically significant overall cognitive benefit in community-dwelling older adults, although findings remain heterogeneous across outcomes and populations [28,29].

Recent trials and reviews in certain disability populations (eg, stroke, Parkinson disease, and multiple sclerosis) indicate that exergaming interventions can be feasible and may improve physical activity participation and related outcomes when appropriately adapted to users’ functional abilities and delivered with adequate support and progression [18,19]. For example, a 2026 pilot randomized controlled trial in people with moderate to advanced Parkinson disease reported positive acceptability in 84% of participants, attendance adherence of 99.7%, and no intervention-related adverse events, with exploratory findings suggesting possible benefits for health-related quality of life and loneliness [30]. However, variability in intervention dose, outcome selection, and accessibility of commercial hardware remains a major implementation barrier. These challenges underscore the need to evaluate adapted interfaces and delivery models that accommodate users who require seated play or have limited limb function.

Key factors influencing the effectiveness of AVGs include player enjoyment, engagement, and perceived exertion. Enjoyment and engagement have been identified as mediators between gameplay and energy expenditure, suggesting that more enjoyable and engaging games may lead to higher levels of physical activity [31]. Additionally, perceived exertion during AVG play is often lower than actual exertion, indicating that players may not fully recognize the physical benefits they are attaining [32].

Research on physical activity for individuals with mobility impairments is increasing and has shown that AVGs present viable and enjoyable means for them to engage in physical activity [7,33-35]. A growing body of literature indicates that people with physical disabilities can increase their energy expenditure during AVG play, including those with mobility impairments such as cerebral palsy, spinal cord injury, stroke, and other neurological conditions [33,34,36,37]. Furthermore, AVGs can mitigate common environmental barriers to conventional exercise such as facility and program access and transportation. At the same time, recent adaptive gaming research suggests that participation barriers extend beyond device availability and include access to centralized information resources, financial assistance, professional support for setup and configuration, and opportunities to try equipment before adoption. These broader implementation factors are important when considering whether adapted gaming approaches can be translated into routine home, community, or clinical use for people with physical disabilities [38]. Since AVG devices are relatively affordable, they also hold promise as a scalable solution for promoting higher levels of physical activity and fitness among people with disabilities.

Despite these findings, an ongoing limitation in the translation of exergaming to adults with mobility impairments is that many commercial systems rely on standing balance, rapid lower-extremity stepping, or controllers that are not feasible for seated users or those with limited limb function. Consequently, even as exergaming evidence grows, fewer studies evaluate interventions using adapted interfaces designed specifically for seated play and for people with more severe mobility limitations, creating a gap between what is theoretically promising and what is practically accessible.

To address this need, our research and engineering team has developed AVG controllers that are accessible to people who are unable to stand for long periods, cannot stand on a small platform due to poor balance or extreme obesity, cannot safely stand on a small platform due to balance limitations or body size, or who primarily use a wheelchair for mobility [33,37,39-42]. Most recently, we developed the GAIMplank controller and evaluated its usability among people with mobility impairments [42]. During usability testing, a sample of 21 adults was able to successfully access and play a series of standard PC video games using trunk or body movements to produce gameplay action. Participant usability scores and qualitative feedback indicated above-average usability for the GAIMplank system. In addition, participants enjoyed the activity and reported gameplay as light-to-moderate intensity activity.

Although these findings support the GAIMplank’s usability and perceived exertion during single-session gameplay, usability alone does not establish whether an adapted interface can be delivered as an intervention over time or whether it will be acceptable and engaging across repeated sessions. Building from these promising usability results of the GAIMplank controller, the next step is to evaluate whether a multisession intervention using this adapted interface is feasible and acceptable in adults with mobility impairments, and whether participants report adequate perceived exertion alongside enjoyment and engagement, key determinants of sustained participation. Therefore, the aim of this concurrent mixed methods single-arm pilot study was to evaluate a 6-week AVG intervention delivered twice weekly to adults with mobility impairments by assessing quantitative outcomes (perceived exertion, engagement, and enjoyment) and qualitative perspectives regarding the intervention’s acceptability, appropriateness, and feasibility.

## Methods

### Research Design Overview

This study used a concurrent mixed methods single-arm pilot design [43]. In this design, quantitative (feasibility, enjoyment, and engagement) and qualitative (participant feedback and staff observations) data were collected simultaneously and integrated to provide a comprehensive evaluation of the GAIMplank intervention. The rationale for using a mixed methods approach was to triangulate objective descriptive statistics with subjective participant experiences to assess the protocol’s feasibility and acceptability among adults with mobility limitations.

### Researcher Description

Our team comprises experts in exercise physiology, health services research, and engineering. Two of the researchers (CJM and SM) are trained in qualitative and mixed methods research. One researcher was involved in data collection (CJM), and three (CJM, LAM, and SM) were involved in data analysis and integration of the results. The team has published a few other mixed methods studies.

### Inclusion and Exclusion

Inclusion criteria were (1) being aged 18 years or older, (2) self-reported physical disability that limits mobility, and (3) ability to communicate in English. Exclusion criteria were (1) significant impairment in visual acuity that prevents following video games on a 52″ television screen, (2) body weight >400 lbs, (3) cognitive or linguistic problems with understanding instructions or filling in self-administered outcome measures in English as determined by a score of <17 on the telephone version of the Mini-Mental State Examination, (4) any other conditions that would limit ability to play video games, (5) cardiovascular disease event within the past 6 months, (6) severe pulmonary disease or renal failure, and (7) ongoing exacerbation of a health condition.

### Participant Recruitment

#### Recruitment Process

Both quantitative and qualitative data were collected from the same participants. Flyers were distributed to local organizations serving adults with disabilities. Individuals who were interested in participating called the number listed on the flyer to speak with the recruitment coordinator, who provided a brief overview of the project and a series of screening questions to determine eligibility. In addition, phone calls were made to previous research participants who had agreed to be contacted for other studies. If deemed eligible, the first laboratory visit was scheduled.

#### Sampling

This was a pilot feasibility and acceptability study of the adapted GAIMplank. A convenient sampling of 6 participants was recruited. Of the 6 participants who consented and started the study, a total of 4 completed the intervention.

#### Data Collection Location

Data collection took place in the RecTech Exercise Science and Technology Laboratory at the University of Alabama at Birmingham.

### Ethical Considerations

This study was conducted in accordance with ethical principles for research involving human participants. The study protocol was reviewed and approved by the University of Alabama at Birmingham Institutional Review Board (approval IRB-300009049). The study was classified as nonexempt human subjects research involving no more than minimal risk, in accordance with applicable federal regulations. All participants provided informed consent prior to participation in the study. Participants were informed about the purpose of the study, study procedures, potential risks and benefits, voluntary participation, and their right to withdraw at any time without penalty. Participant privacy and confidentiality were protected throughout the study. All data were deidentified prior to analysis and stored on a secure, password-protected server accessible only to authorized study personnel. No direct identifiers were included in the dataset, and results are reported in aggregate to prevent identification of individual participants. No images or supplementary materials included in this manuscript contain identifiable information about individual participants. Participants received compensation of up to US $125 for participating in the study. The money was distributed in the form of gift cards as follows: upon completion of visit 2 (US $25) and at the end of the 6 weeks after completion of the assessments (US $100).

### Adapted Gaming Board (GAIMplank) and Game Selection

**Figure 1:**
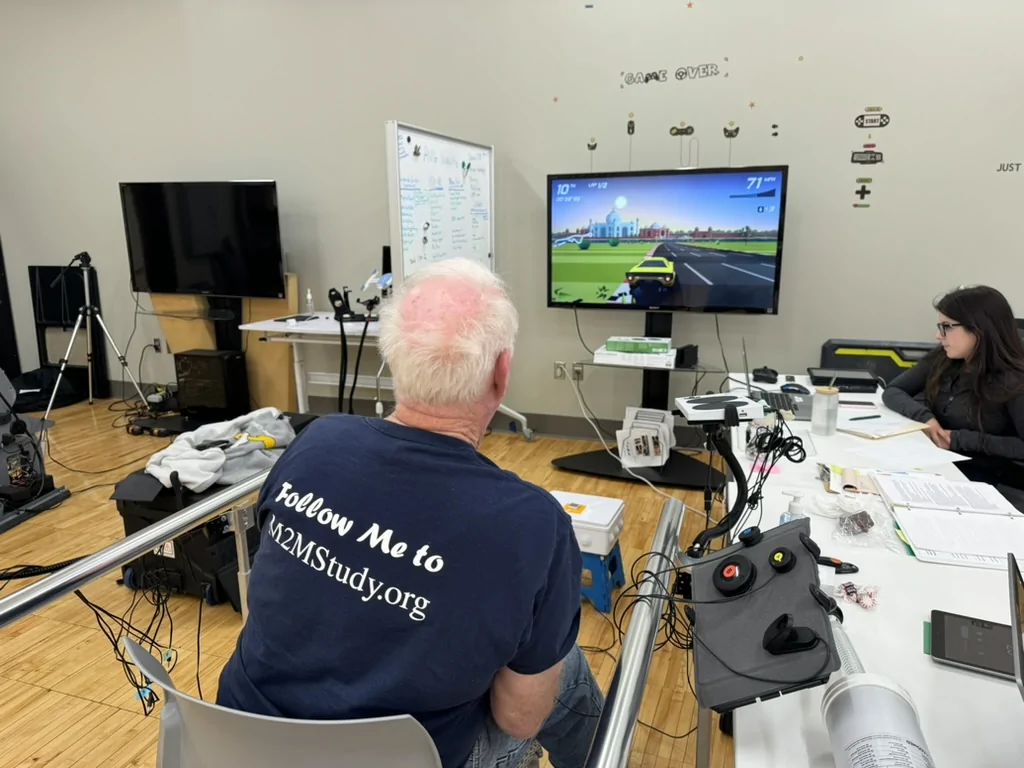
Participant playing an active video game using the GAIMplank. Participant is seated in a chair on top of the GAIMplank using his body (leaning right and left) to control movement of the car on the screen, holding a variable trigger in his hand for acceleration.

Our engineering design and development team, with the Rehabilitation Engineering Research Center on Interactive Exercise Technologies and Exercise Physiology for People with Disabilities, previously built a proof-of-concept wheelchair-accessible adapted gaming controller for the Nintendo Wii home video game console [39]. Our research team tested its usability rating compared to the commercial off-the-shelf balance board controller provided with the Wii console [40]. From that, we developed a new iteration of the adapted board, now called the GAIMplank, and tested its usability [42]. The GAIMplank was designed as a wheelchair-accessible AVG controller for PC-based gameplay and includes a balance board platform with roll-on access, height-adjustable handrails, embedded load cells, and custom electronics that translate shifts in the player’s center of balance into game inputs. As with the previous version, the GAIMplank was mapped to operate as a typical joystick controller. Leaning movements of the trunk created corresponding action in the game (ie, lean forward, move character forward or up, lean right, and move right; Figure 1). External buttons to activate actions such as jump or shoot could be configured in various formations on a flexible arm within the player’s reach, held by the player, or placed in the player’s lap. A trigger button used for acceleration or shooting could be held in the hand. Full technical details have been reported previously [42].

Activity guidelines developed a priori by the research team were used to inform video game task selection and progression. The final list of games for use during the intervention is presented in Table 1.

**Table 1:** List of the off-the-shelf video games played during the intervention, including a short description of each game, trunk movements required for gameplay, and any external buttons and their action used.

Game	Genre	Game description	Trunk-leaning movements required	External buttons used
*Feather*	Flight, exploration	Relaxing flight to explore a scenic natural landscape	All directions	None
*Flower*	Adventure	Serene journey as a flower petal navigating landscapes in the wind	All directions	Button to accelerate
*Bezier*	Arcade, shooter	Fast-paced shooter with neon visuals	All directions	Button to shoot
*Space Invaders Extreme*	Arcade, shooter	Classic arcade-style shooting game	Left, right	Button to shoot
*Explorers*	Adventure	Exploration and resource management as an adventurer	All directions	None
*The Lost Night*	Adventure, shooter	Atmospheric adventure evading threats and making key decisions	Right, left, up, down	Button to shoot
*Let’s Go Nuts*	2D platformer, action	Jump, avoid danger, and collect nuts while leveling up	Left, right	Button to jump
*Omeganaut*	Shooter	Control a spacecraft battling enemies across levels	All directions	Button to shoot
*Stardust Galaxy Warriors*	Shooter, action	Soar in space using weapons to combat enemy ships and asteroids	All directions	Up to 4 buttons for weapon use
*Shipwreck*	Adventure, action	Retro adventure with island exploration and enemy fighting	All directions	Button to use objects, attack, interact
*Galaga*	Arcade, shooter	Space shooter, defend against alien enemies, rescue captured ships, and boost firepower	Left, right	Button to shoot
*Sheepy*	2D platformer	Guide a sheep jumping over obstacles and shooting lollipops	Left, right	Up to 3 buttons to run, jump, shoot
*Horizon Chase Turbo*	Racing, sports, arcade	Arcade racing games with various cars and tracks to select from	Left, right	Variable trigger for car acceleration
*Sonic Mania*	2D platformer, action	Fast-paced adventure, run, jump, collect golden rings, and beat enemies	Left, right	Button to jump and roll

### Participant Visits

During the first visit to the laboratory, informed consent was obtained, baseline assessments were collected, and the gameplay sessions were scheduled. Participants then attended individual gameplay sessions supervised by a member of the research team 2 times weekly for 6 weeks, for a total of 12 sessions. This schedule was selected to balance safety, standardization, repeated exposure to the adapted gaming system, and participant burden. It also provided adequate exposure for a pilot study while remaining manageable for adults with mobility impairments who may experience fatigue, transportation, or other participation barriers. The intervention protocol included various PC video games that were compatible with the GAIMplank. For each gameplay session, participants played seated in a standard 4-legged chair or their wheelchair. During the first 4 visits, participants played 5 minutes of 3 or 4 preselected games to expose them to the various game options available for subsequent sessions.

As the weeks progressed, participants advanced to longer continuous bouts of activity until they achieved approximately 50 minutes of activity. This target duration was selected pragmatically to provide a meaningful amount of active gameplay each week, considering broader physical activity recommendations, while remaining feasible and tolerable for participants with mobility impairments in a supervised laboratory setting. Although this dose was not intended to meet weekly physical activity guidelines, it supported progressive engagement while accounting for participant tolerance and the need to monitor fatigue and safety. The research team started each game at the basic or beginner level and advanced it to the next level as dictated by game progression and participant preferences. The gameplay sessions were adjusted by the research team based on participant needs with continued progression and balance of activities. Progression of activities and gameplay duration was guided by participant safety, independence, use of appropriate movement strategies, level of engagement, and fatigue.

After the last gameplay session, participants completed an acceptability, appropriateness, and feasibility survey as well as a semistructured interview conducted by a member of the research team. The interviews focused on topics related to participants’ experience of and engagement with the adapted gaming board.

### Data Collection

The mixed methods concurrent design procedures are presented in Textbox 1.

**Mixed methods concurrent design procedures.**

**Timing of quantitative and qualitative data collection, analysis, and integration throughout the 6-week active video gaming intervention**

**Quantitative data**

- Baseline
  Participant demographics
  Patient-Reported Outcomes Measurement Information System Physical Function—Short Form 20a
  Multidimensional Outcome Exercise Expectations Scale
  Physical Activity Scale for Individuals With Disabilities
- Gameplay sessions 1, 5, and 12
  Physical Activity Enjoyment Scale
  Exergame Enjoyment Questionnaire
- Each gameplay session
  Game enjoyment rating scale
  Game engagement rating scale
  Game rating of perceived exertion
- Postintervention
  Acceptability, appropriateness, and feasibility of the active video gaming intervention survey

**Qualitative data**

- Each gameplay session
  Gameplay experience
- Postintervention
  Participant feedback interviews

### Quantitative Measures

#### Participant Characteristics

Demographics (baseline) data included age, sex, race, primary mobility impairment, and assistive device use for mobility.

#### Patient-Reported Outcomes Measurement Information System Physical Function—Short Form 20a (Baseline)

The Patient-Reported Outcomes Measurement Information System Physical Function—Short Form 20a assesses an individual’s self-reported capability to perform physical activities [44]. It measures various aspects of physical function, including mobility (eg, walking and running), upper extremity function (eg, reaching and grasping), and activities of daily living (eg, bathing and dressing). It is standardized and validated for a variety of populations, including those with chronic conditions and disabilities. The form includes 20 questions, each scored from 1=without any difficulty to 5=unable to do so. Raw scores were converted to a *T* score using the Patient-Reported Outcomes Measurement Information System scoring manual. The *T* score is standardized (mean 50, SD 10), with higher scores indicating better physical functioning and lower scores reflecting limitations or difficulties in physical activities. Severity of impairment is indicated as mild (40-45), moderate (30-40), or severe (below 30).

#### Physical Activity Scale for Individuals With Disabilities (Baseline)

The Physical Activity Scale for Individuals With Disabilities (PASIPD) is a self-report questionnaire designed to assess physical activity levels among individuals with disabilities [45]. It evaluates the frequency, duration, and intensity of various activities over the past 7 days, including leisure, household, and work-related activities. The PASIPD includes 13 items that cover a variety of physical activities, such as walking or wheeling, light and heavy household chores, and recreational activities. The PASIPD score is calculated by multiplying the time spent in each activity (hours per week) by its metabolic equivalent value and then summing these products across all activities. Scores range from 0=no activity to over 100 metabolic equivalent/hours per day, which indicates very high levels of physical activity.

#### Multidimensional Outcome Exercise Expectations Scale (Baseline)

The Multidimensional Outcome Exercise Expectations Scale (MOEES) is a validated 15-item tool [46] used to measure an individual’s expectations about the benefits of engaging in regular exercise. The scale evaluates 3 key dimensions of exercise outcome expectations including physical (n=6 items), social (n=4 items), and self-evaluative (n=5 items) benefits. Items are rated on a 5-point Likert scale, in which 1 represents strongly disagree and 5 represents strongly agree. Each subscale was scored by summing the numerical ratings for each response, with a higher score on any subscale indicating a stronger belief in the positive outcomes associated with exercise related to that specific dimension. An overall total score was also computed by summing all items, with higher scores (range 15-75) indicating stronger positive expectations for exercise outcomes.

#### 16-Item Physical Activity Enjoyment Scale (Gameplay Sessions 1, 5, and 12)

The Physical Activity Enjoyment Scale (PACES) was designed to measure an individual’s enjoyment of physical activity. The 16-item version was used for this study [47]. Each of the items on PACES was rated on a 5-point Likert scale from 1=strongly disagree to 5=strongly agree. In total, 7 of the items were negatively worded and reverse-scored, and then, all items were summed for a total score ranging from 16 to 80. A higher score indicated greater enjoyment of the AVG physical activity.

#### Exergame Enjoyment Questionnaire (Gameplay Sessions 1, 5, and 12)

The 20-item Exergame Enjoyment Questionnaire (EEQ) was designed to measure enjoyment of exergames [48]. In total, 8 items were negatively worded and reverse-scored, and then, all items were summed for a total score ranging from 20 to 100. A higher score indicated greater enjoyment of the exergames.

#### Rating of Perceived Exertion During Gameplay (Each Gameplay Session)

Participants provided a rating of perceived exertion (RPE) score after each game using the OMNI Scale of Perceived Exertion [49], with a score ranging from 0=extremely easy to 10=extremely hard.

#### Game Enjoyment and Engagement Rating Scales (Each Gameplay Session)

Participants rated their level of enjoyment of each game using a 10-cm visual analog scale from 0=least enjoyment to 10=most enjoyment. Participants also rated their level of engagement during each game using a 10-cm visual analog scale from 0=least engaged to 10=most engaged.

### Implementation Outcomes (Postintervention): Acceptability, Appropriateness, and Feasibility of the AVG Intervention

At the completion of the intervention, participants completed the Acceptability of Intervention Measure (AIM), Intervention Appropriateness Measure (IAM), and Feasibility of Intervention Measure (FIM) [50]. Each of the 3 measures was comprised of 4 questions rated on a 5-point scale from 1=completely disagree to 5=completely agree. A score for each measure was computed by averaging the responses, with scores ranging from 1 to 5 (higher scores indicate better perceived acceptability, appropriateness, and feasibility).

### Qualitative Data Sources

#### Gameplay Observations (Each Gameplay Session)

During each gameplay session, the research staff maintained field notes on observations, such as GAIMplank responsiveness, specific participant assistance needs, and participants’ spontaneous verbal feedback about their experience.

#### Participant Feedback Interviews (Postintervention)

The research staff conducted semistructured interviews after participants completed the intervention. A series of questions was used to guide the conversation, and directed prompts were used as needed. The interviews were audio-recorded, transcribed verbatim, and checked for accuracy against the audio recordings.

Several differences between the original trial registration, institutional review board–approved protocol, and final assessment battery should be noted. The original trial registration listed the International Physical Activity Questionnaire for People with Disabilities as the physical activity outcome measure; however, prior to intervention data collection, the research team replaced it with the PASIPD to maintain consistency with related studies and better align with the study population. This change was approved by the institutional review board, but the ClinicalTrials.gov record was not updated at that time due to an administrative oversight. In addition, the Short Form 36 Health Survey (version 2) was included in earlier study documentation but was not collected to reduce participant burden. RPE, engagement or enjoyment ratings, AIM/IAM/FIM, EEQ, and MOEES were included in the institutional review board–approved protocol and collected as part of planned study procedures but were not prospectively added to the ClinicalTrials.gov registration. The ClinicalTrials.gov record has since been updated to reflect the final outcome measures.

### Data Analysis and Integration

#### Quantitative Data Analysis

Quantitative data were analyzed using descriptive statistics. Demographic and baseline characteristics of participants were summarized using means and medians for continuous variables and frequencies for categorical variables. Surveys were scored using the standard scoring protocols for each instrument.

#### Qualitative Data Analysis

Qualitative data were analyzed using thematic analysis. Two coders (SM and LAM) coded the data and met to resolve coding discrepancies. Codes were generated from recurring themes and patterns related to participants’ experiences during the intervention. Codes were grouped into broader themes that captured key aspects of participants’ experiences, such as perceived exertion, enjoyment, engagement, and overall acceptability of the intervention.

#### Mixed Methods Integration of Quantitative and Qualitative Data

Quantitative and qualitative findings were integrated to provide a comprehensive understanding of participants’ experiences. A joint display was used to compare and contrast quantitative and qualitative results, highlighting areas of convergence and divergence. The integration of quantitative and qualitative data provided a holistic view of the intervention’s impact. Quantitative data offered objective measures of perceived exertion, enjoyment, and engagement, while qualitative data provided deeper insights into participants’ subjective experiences. This mixed methods approach allowed for a more nuanced understanding of the feasibility, acceptability, and potential benefits of the AVG intervention for adults with mobility impairments.

### Validity, Reliability, and Methodological Integrity

#### Quantitative Validity and Reliability

Standardized tools (Patient-Reported Outcomes Measurement Information System Physical Function—Short Form 20a, MOEES, and PASIPD) provided construct validity for baseline and activity assessments. The real-time data collection using the OMNI scale and visual analog scales ensured consistent, immediate measurement of the gameplay experience.

#### Qualitative Methodological Integrity

Trustworthiness was maintained through verbatim transcription of all interviews and verification against the audio recordings. Methodological rigor was maintained through a systematic thematic analysis of staff notes and participant interviews to identify recurring patterns. While the thematic saturation was not reached due to the small sample size, the findings were grounded in direct participant evidence.

#### Mixed Methods Integrity and Integration

The integration of the concurrent mixed methods datasets was formalized through a joint display, which enabled direct comparison of quantitative scores with thematic feedback. This process ensured convergent validity, as qualitative quotes were used to contextualize and explain the quantitative findings.

## Results

### Overview

In total, 24 individuals were contacted during recruitment and screening. A total of 18 did not proceed to enrollment because they did not respond, declined participation, or were unavailable, resulting in a final enrolled sample of 6 participants. The average age of the participants was 65.2 (SD 12.4) years, and most had experienced a stroke. All but one person used an assistive device for mobility. Additional participant details are presented in Tables 2 and 3. Patient-Reported Outcomes Measurement Information System Physical Function scores indicated a moderate level of impairment in physical function for 4 participants, 1 participant with mild impairment, and 1 participant with severe impairment (AVG-06) who subsequently dropped out. MOEES scores indicated that all participants had strong positive beliefs regarding the value of exercise. The daily physical activity level of individuals ranged from low to moderate, as reported on the PASIPD scale. Participant AVG-02 dropped out after the second gameplay session, and AVG-06 after the sixth gameplay session, both due to health reasons.

**Table 2:** Participant (N=6) demographics including age, sex, race, mobility impairment, and assistive devices used for mobility.

ID	Age	Sex	Race	Mobility impairment	Assistive device
AVG-01	44	Female	African American	Stroke	Wheelchair
AVG-02	57	Female	White	Stroke	None
AVG-03	72	Male	White	Stroke	Cane
AVG-05	68	Male	White	Stroke	Cane
AVG-06	75	Male	White	Parkinson disease	Wheelchair
AVG-07	75	Male	African American	Stroke-like symptoms	Cane

**Table 3:** Self-report survey scores collected at baseline for physical function (PROMIS PF20a^a^), outcome expectations for exercise (MOEES^b^) subscale and total scores, and daily physical activity (PASIPD^c^) are listed for each participant^d,e^.

ID	PROMIS PF20a (*T* score)	MOEES, physical (5 to 30)	MOEES, social (5 to 20)	MOEES, self-evaluative (5 to 25)	MOEES, total (15 to 75)	PASIPD (0 to 100+)
AVG-01	33.1	17	13	21	51	3
AVG-02	39.3	29	16	23	68	40
AVG-03	33.9	25	12	20	57	52
AVG-05	37.2	30	20	25	75	18
AVG-06	21.7	24	13	18	55	6
AVG-07	43.7	28	16	25	69	51.5

^a^PROMIS PF20a: Patient-Reported Outcomes Measurement Information System Physical Function—Short Form 20a.

^b^MOESS: Multidimensional Outcome Exercise Expectations Scale.

^c^PASIPD: Physical Activity Scale for Individuals With Disabilities.

^d^The Patient-Reported Outcomes Measurement Information System scores are reported as standardized *T* scores (mean 50, SD 10), with higher scores indicating better physical functioning with mild impairment score of 40-45, moderate 30-40, and severe <30. For each MOEES score, a higher score indicates a stronger belief in the positive outcomes associated with exercise in a specific dimension. The PASIPD score is calculated by multiplying the time spent in a variety of physical activities (hours per week) by its metabolic equivalent value and then summing across all activities. Scores range from 0 (no activity) to over 100 metabolic equivalent/hours per day (very high).

^e^PROMIS PF20a: median 70 (IQR 61-76); MOEES, physical: median 27 (IQR 24-29); MOEES, social: median 15 (IQR 13-16); MOEES, self-evaluative: median 22 (IQR 20-25); MOEES, total: median 63 (IQR 55-69); PASIPD: median 29 (IQR 6-52).

Participant data for each week are shown in Table 4. As noted, minutes of gameplay increased each week. The number of different games played per week ranged from 2 to 8 and declined in the later weeks, as participants found the games they liked and stuck with them. Recorded RPE scores were in the moderate intensity exercise range for the majority of players, who reported above-average enjoyment most weeks. All participants reported high (≥8) engagement each week.

**Table 4:** Minutes and number of games played per week for each participant, along with weekly median self-report ratings of perceived exertion (RPE), engagement, and enjoyment (0-10 rating scale)^a^.

Participant ID and week		Total time played (minutes)	Games played, n	RPE (0-10), median (IQR)	Enjoyment (0-10), median (IQR)	Engagement (0-10), median (IQR)
**AVG-01**						
	1	39	7	—^b^	8 (5-10)	10 (9-10)
	2	40	8	—	7 (4-8)	10 (9-10)
	3	65	8	1 (0-2)	9 (3-10)	9 (8-10)
	4	85	5	1 (0-1)	10 (9-10)	10 (10-10)
	5	96	4	0 (0-2)	10 (10-10)	10 (10-10)
	6	102	4	2 (1-2)	10 (10-10)	10 (10-10)
**AVG-02**						
	1	40	7	2 (1-3)	8 (7-10)	9 (8-10)
**AVG-03**						
	1	40	7	4 (3-5)	9 (5-10)	9 (9-10)
	2	40	7	5 (5-5)	9 (7-10)	10 (10-10)
	3	73	7	6 (4-6)	10 (9-10)	10 (10-10)
	4	85	5	6 (5-6)	10 (9-10)	10 (9-10)
	5	92	6	6 (5-7)	10 (9-10)	10 (10-10)
	6	90	5	6 (6-7)	10 (7-10)	10 (9-10)
**AVG-05**						
	1	40	7	8 (6-8)	4 (3-5)	8 (8-8)
	2	40	7	6 (6-7)	8 (5-9)	9 (8-10)
	3	75	4	7 (6-8)	5 (4-7)	8 (7-9)
	4	75	4	7 (6-8)	6 (5-7)	8 (7-9)
	5	96	3	7 (6-8)	7 (4-7)	8 (6-9)
	6	66	4	8 (6-8)	6 (5-8)	8 (8-9)
**AVG-06**						
	1	40	7	6 (6-6)	5 (4-6)	8 (7-8)
	2	39	7	5 (3-6)	6 (3-6)	8 (6-8)
	3	68	3	6 (6-6)	8 (4-9)	8 (6-9)
**AVG-07**						
	1	40	7	4 (3-5)	9 (7-9)	9 (9-10)
	2	40	8	5 (4-5)	10 (10-10)	10 (9-10)
	3	69	2	5 (4-6)	10 (10-10)	10 (10-10)
	4	85	3	5 (5-6)	10 (10-10)	10 (10-10)
	5	95	2	5 (5-6)	10 (10-10)	10 (10-10)
	6	103	2	6 (5-6)	10 (10-10)	10 (10-10)

^a^Higher RPE scores indicate greater perceived exercise intensity, whereas higher enjoyment and engagement reflect more positive gameplay experiences.

^b^Not available.

Rankings of the most-played games and the average RPE, enjoyment, and engagement for each game are presented in Table 5. The top 3 games based on total minutes played across participants across sessions were *Space Invaders*, *Let’s Go Nuts*, and *Horizon Chase Turbo*, representing 3 different game genres, shooter, platformer, and racing, respectively. Participants rated all 3 games high for both enjoyment and engagement and reported perceived exertion as somewhat hard, suggesting moderate-intensity exercise. The least played games were those that required movement in all directions. Although participants rated several games as not highly enjoyable, they still reported that the games were engaging.

PACES scores indicated a moderate level of enjoyment at each time point across participants, with a range of 40 to 50 (Table 6). For all participants, EEQ scores were above average at each time point, ranging from 54 to 86.

**Table 5:** Total gameplay minutes for each active video game across all participants and all intervention sessions, with median self-reported ratings of perceived exertion (RPEs), enjoyment, and engagement (0-10 rating scales) aggregated across participants and sessions for each game.

Game name	Time played (minutes), total	RPE (0-10), median (IQR)	Enjoyment (0-10), median (IQR)	Engagement (0-10), median (IQR)
*Space Invaders*	416	6 (6-7)	10 (7-10)	10 (9-10)
*Let’s Go Nuts*	338	5 (2-6)	10 (9-10)	10 (9-10)
*Horizon Chase Turbo*	323	6 (2-7)	9 (7-10)	10 (9-10)
*Sonic Mania*	207	4 (1-6)	10 (9-10)	10 (9-10)
*Sheepy*	125	3 (2-6)	9 (8-10)	10 (9-10)
*Galaga*	83	6 (4-7)	9 (4-9)	9 (7-10)
*Omeganaut*	66	5 (5-6)	9 (5-10)	10 (8-10)
*Explorers*	65	5 (4-6)	7 (4-9)	9 (8-10)
*Bezier*	60	6 (3-6)	6 (6-8)	8 (8-10)
*Feather*	60	3 (1-7)	5 (4-7)	8 (7-9)
*Flower*	40	3 (1-4)	6 (3-9)	9 (8-9)
*Shipwreck*	27	5 (5-8)	4 (2-8)	9 (6-10)
*Stardust Galaxy*	25	5 (5-6)	8 (6-10)	10 (8-10)
*The Lost Night*	25	5 (4-6)	6 (3-8)	10 (5-10)

**Table 6:** Participants’ self-reported enjoyment scores using the Physical Activity Enjoyment Scale (PACES) and the Exergame Enjoyment Questionnaire (EEQ) following gameplay sessions 1, 5, and 12 of the intervention.

	Gameplay session 1		Gameplay session 5		Gameplay session 12	
	PACES	EEQ	PACES	EEQ	PACES	EEQ
AVG-01	40	82	—^a^	—	42	76
AVG-02	46	78	—	—	—	—
AVG-03	46	81	46	83	47	73
AVG-05	48	74	48	75	52	76
AVG-06	44	54	44	63	—	—
AVG-07	47	86	50	90	48	86

^a^Not available.

Participant ratings of acceptability (AIM), appropriateness (IAM), and feasibility (FIM) of the AVG intervention are reported in Table 7 (AIM: median 5.0, IQR 4.0-5.0; IAM: median 4.1, IQR 3.9-4.3; FIM: median 4.4, IQR 3.8-4.9). Participants agreed or completely agreed that the intervention was acceptable, appropriate, and feasible.

**Table 7:** Participant ratings of acceptability, appropriateness, and feasibility measured using the Acceptability of Intervention Measure (AIM), Intervention Appropriateness Measure (IAM), and Feasibility of Intervention Measure (FIM) collected at the completion of the 6-week intervention^a^.

ID	AIM	IAM	FIM
AVG-01	5	4.25	4.75
AVG-02	—^b^	—	—
AVG-03	5	3.75	3.5
AVG-05	4	4	4
AVG-06	—	—	—
AVG-07	5	4.25	5

^a^Each measure consists of 4 items rated on a 5-point Likert scale, with item responses averaged such that higher scores indicate more favorable perceptions.

^b^Not available.

### Gameplay Sessions: Staff and Participant Comments

#### Overview

**Figure 2:**
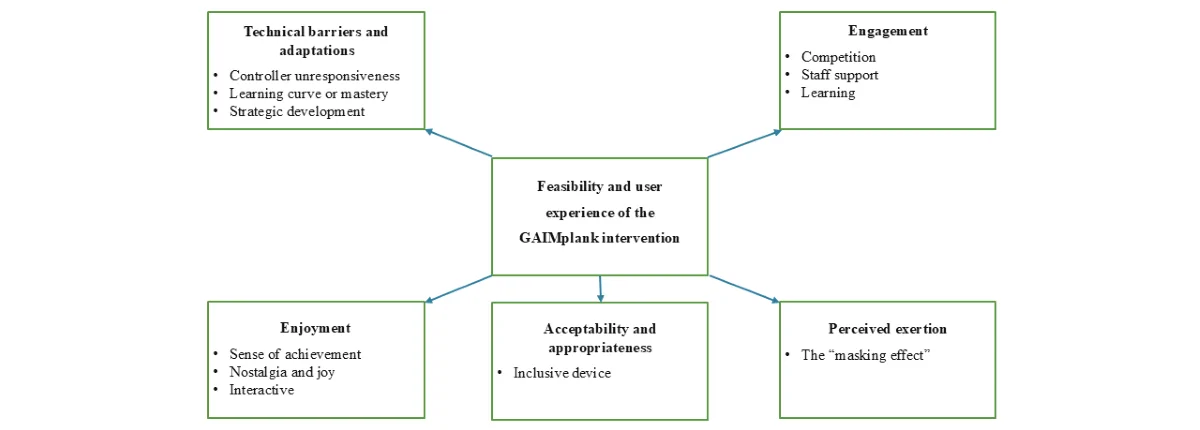
Thematic map.

A summary of staff and participant comments highlights themes that occurred during the gameplay sessions (Figure 2).

#### Technical Issues With the GAIMplank Board

The board often needed to be reset or recalibrated due to unresponsiveness or incorrect movement detection. This was a recurring issue across multiple sessions. There were also consistent reports of the board having a delayed response to participants’ movements, sometimes taking several seconds to register actions. The board often struggled with detecting left and right movements accurately, and there were instances in which it would push participants to one side or fail to recognize movements in specific directions.

#### Participant Frustration and Adaptation

Participants frequently expressed frustration when the board did not respond as expected, mainly when it affected their ability to control their avatars and play the games effectively. Despite initial difficulties, some participants adapted to the board’s quirks over time, improving their gameplay and finding ways to work around the technical limitations.

#### Engagement and Enjoyment

Participants’ enjoyment varied depending on the game and the responsiveness of the board. Some games were more engaging and enjoyable, while others led to frustration due to technical issues. Participants preferred games that were less affected by the board’s technical issues and that matched their interests and abilities. Certain games encouraged more physical movement, which staff and participants reported as a positive aspect. However, the board’s effectiveness in promoting physical activity was inconsistent due to technical problems. Games requiring complex or rapid movements were particularly challenging for participants, often due to the board’s delayed response or inability to register movements accurately.

#### Proactive Problem-Solving

Staff frequently intervened to reset or recalibrate the board, troubleshoot issues, and encourage participants. Staff noted that some games were not well-suited to the board’s capabilities, suggesting a need for better alignment between game design and the adapted controller’s functionality.

These themes emphasize the need to address technical issues with the GAIMplank board to improve the overall experience and effectiveness of AVG interventions for individuals with mobility impairments. Ensuring that equipment performs reliably and providing adequate support during gameplay can enhance participant engagement, enjoyment, and physical activity outcomes.

### Postintervention: Participant Interviews, Experience, and Feedback

#### Overview

Overall, participants had a positive experience using the gaming board, describing it as fun and enjoyable; however, some noted a learning curve when using the board to play some games. Initial frustration gave way to enjoyment as they became more accustomed to the adapted gaming board. Favorite aspects of the 6-week program included the ability to choose games, interaction with others (ie, research staff), and enjoyment of the games themselves. The least favorite aspects were minimal and included the effort of coming to the laboratory. In addition, some participants found gameplay challenging because the board was not responsive to their movements, but they learned how to adapt for gameplay control. Participants generally felt comfortable and challenged using the adapted board. They noted improvement in their ability to use the board and increased comfort as they became more familiar with the system. Participants were unsure about the board’s potential to increase their physical activity but acknowledged its usefulness for individuals with impaired physical abilities. They saw the leaning movements as a form of exercise**.** One participant said he considered getting old games out at home since the study brought back memories. Participant feedback and quotes are organized in the context of the primary outcomes, perceived exertion, enjoyment, and engagement, followed by participant thoughts on feasibility, appropriateness, and acceptability.

#### Perceived Exertion: Effortless Physical Activity

Participants highlighted that adapted video games not only stimulated them mentally, but that the body motion the games required challenged them physically. The competitive nature of games like *Let’s Go Nuts* and *Space Invaders* motivated players to engage in different movements, leading to physical exertion without conscious effort because they were engrossed in gameplay. One participant reported postsession soreness, due to the physical demands of gameplay. Overall, participants saw the AVGs as effective tools for physical activity (Textbox 2).

**Participant experiences of physical activity and exertion during gameplay.**

“Let’s go nuts and space invaders that challenge me mentally as well as ... physical exercise with body motion” [AVG-07].

“... after the session I was physically sore. ... I’m 90% sure that the soreness was from doing the exercising during the games, but I wasn’*t* 100% sure if it was that or it was my disability because the stroke is left side ... but I’m pretty sure I was sore from moving around quite a bit” [AVG-05].

“You know, especially for people that are worse off than me, some people I’m sure that movement is good exercise, you know, I mean, it’s exercise for me too. But, obviously I can do bigger and better things, but that’s pretty cool that that’s you are getting exercise out of playing the games” [AVG-05].

#### Enjoyment: Achievement and Interaction Over Frustration

Participants reported significant enjoyment when playing adapted video games, highlighting their engaging quality. Initial frustrations with response times diminished over time, leading to increased enjoyment. The games fostered a sense of achievement, nostalgia, and joy in participants, who anticipated that gameplay would be enjoyable for others with disabilities (Textbox 3).

**Fun, challenge, and enjoyment during gameplay.**

“Wonderful, lots of fun, always enjoyable” [AVG-01].

“Enjoyment with interaction ... Enjoyable. Lots of fun. ... Have thought about getting old games out at home since the study brought back memories” [AVG-03].

“Was frustrated not being able to move quickly enough, other times not responding as quickly as I wanted it to. But I enjoyed it as we went on, I got better. So it really is something I think people would enjoy, people like me with disabilities” [AVG-05].

#### Engagement: Competitive and Supportive Environment

Participants demonstrated gameplay engagement through factors such as competitive motivation, learning, a sense of accomplishment, and staff support. This engagement shaped their connection to the AVG intervention. Interaction with and support from research staff fostered a motivating environment for participation. The competitive elements of gameplay significantly enhanced commitment among individuals with a competitive nature. Progress in learning, from overcoming challenges to strategic development and improved performance, sustained engagement (Textbox 4).

**Competition, learning, and mastery sustained engagement.**

“Didn’t seem like playing for long due to having fun” [AVG-03].

“My favorite no doubt about this is ‘Let’s Go Nuts’ and the reason is the competitive nature of the game. You know, being a coach as many years as I coach, you know that competitive nature haven’t left me yet, even though I left the coach in the room, the nature still within me. So anytime I get into a competitive environment it just motivates me” [AVG-07].

“Learning experience, it was frustrating, but fun and more fun than frustrating. Once I got better. Once I got the concept and started getting, you know, it’s like the more you play a game, the better you get. That’s the way it was. The more I did it, the better I enjoyed it and the better I became. At first it was frustrating because I wasn’t able to, like, keep the car on the track or shoot. Things are I wanted to go because I’m pushing the button but not leaning. And then I realized I’m not leaning. You know, so I got better and I enjoyed that. Once I got that part down and got my movements a little better” [AVG-05].

“First you have to analyze the game you plan. And realize that we have to develop a strategy for tech. You just can’t go out” [AVG-07].

#### Acceptability, Appropriateness, and Feasibility: Inclusive Device

Participants expressed a general sense of acceptability toward the adapted gaming device, highlighting feelings of accomplishment and the potential for continuous improvement with practice. Additionally, participants emphasized the device’s usefulness for individuals with varying abilities. This inclusivity enhances its acceptability, as it provides an accessible way for individuals to engage in meaningful physical activity. Finally, the device offered enjoyment, with participants expressing hope that others, regardless of ability, would derive as much satisfaction from it as they did.

You feel sense of accomplishment. Made me feel like I was getting better and it’s something that if I practice I could do even better so. You know, it was OK.

AVG-05

Participants recognized the gaming device’s suitability for those with mobility limitations, highlighting the enjoyment it produced and its accessibility for individuals with disabilities. One participant noted that while the physical activity aspect was valuable, the mental benefits—such as improved focus, distraction from personal challenges, and emotional enjoyment—may have had an even greater impact. Participants also noted that the game helped shift attention from their condition to the present activity.

I think this game is extremely beneficial to people who are stationary with their legs. Because not only will it entertain them mentally, but will entertain them physically too. I think the mental exercise will provide more for them than the physical. It’ll get their mind focused off their condition, and focused on enjoyment. Right. It’s distracting with you from what you’re going through to what you’re doing.

AVG-07

Participants’ feedback suggests that the adapted gaming board is a feasible tool for increasing physical activity among individuals with disabilities. Its enjoyable and engaging nature makes it easier for users to stay active without feeling burdened by the idea of exercise. One participant noted that the fun aspect made time pass quickly, indicating that the games can sustain an individual’s attention and promote consistent activity. Overall, the gaming board’s adaptability and engaging design make it a feasible and effective option for promoting physical activity among people with disabilities.

Enjoyable. Lots of fun. Didn’t seem like playing for long due to having fun ... Useful for those who have more limited abilities.

AVG-03

So it really is something I think people would enjoy, people like me with disabilities ... You know, especially for people that are worse off than me, some people I’m sure that movement is good exercise, you know, I mean, it’s exercise for me too. But, obviously I can do bigger and better things, but that’s pretty cool that that’s you are getting exercise out of playing the games.

AVG-05

Table 8 presents the integrated findings, which show strong convergence between the quantitative and qualitative data analyses across all key intervention outcomes. In the case of perceived exertion, the quantitative median RPE of 6, indicating moderate-intensity exercise, was contextualized by the qualitative theme of “effortless physical activity.” Participant described a “masking effect,” in which competitive gameplay and mental focus shifted attention away from physical effort, making exercise a byproduct of play. Similarly, high quantitative ratings for enjoyment (9) and engagement (10) aligned with qualitative reports that mastery of adapted controls and positive staff interactions effectively outweighed initial technical frustrations. The implementation outcomes for acceptability, appropriateness, and feasibility were corroborated by participant feedback, which identified the GAIMplank as a highly suitable and inclusive device for individuals with varying mobility levels. The synthesis underscores that the AVG intervention was both physically engaging and enjoyable and was well-received by participants to stay active.

**Table 8:** Results: joint display integrating quantitative and qualitative findings across key intervention outcomes.

Outcome	Quantitative	Qualitative theme	Qualitative quotes	Integration
Perceived exertion	Median gameplay RPE^a^ across participants was 6 (on 0-10 rating scale)	Effortless physical activity: Exercise occurred as a byproduct of gameplay competition.	“... you’re getting more exercise than you feel like as you’re not focusing on the exercise as much as you were on the competition. And when I left there, someday I feel like down, I mean a mile I ran, but I’m not conscious of what I’m doing ... I don’t know how they measure how much you got of it, but if you can measure it, you’ll realize you got more out of it than you” [AVG-07].	Results converge: Competitive gameplay masked physical effort, allowing moderate exertion without the conscious burden of exercise.
Enjoyment	Median gameplay enjoyment was 9 (on 0- to 10-point scale)	Achievement and interaction over frustration: Mastery of the adapted controls led to joy and nostalgia.	“Enjoyment with interaction ... Enjoyable. Lots of fun. Didn’t seem like playing for long due to having fun ... Have thought about getting old games out at home since the study brought back memories” [AVG-03].	Results converge: High enjoyment score reflects the positive experiences of gameplay, which surpasses the initial technical hurdles and “learning curve.”
Engagement	Median gameplay enjoyment was 10 (on 0-10 rating scale)	Competitive and supportive environment: Mental strategy and staff interaction led to deep engagement.	“Didn’t seem like playing for long due to having fun” [AVG-07]. “My favorite no doubt about this is Let’s Go Nuts ... the competitive nature of the game ... motivates me” [AVG-07]. “Meeting y’all (staff), interacting with new people” [AVG-05].	Results converge: The perfect engagement score aligns with participants feedback on positive engagement facilitated by encouraging research staff and strategic demands by the gameplay.
Implementation outcomes (acceptability, appropriateness, and feasibility)	Agreed or completely agreed (AIM^b^, IAM^c^, and FIM^d^)	Inclusive device: Perceived as highly suitable for varying mobility levels and mental distraction.	“Useful for those who have more limited abilities” [AVG-03]. “I think its game is extremely beneficial to people who are stationary ... It will get their mind, focus on condition, and focus on enjoyment” [AVG-07].	Results converge: The adapted board provided an acceptable, appropriate, and feasible means of engaging in physical activity. The intervention provided an accessible and enjoyable way for persons with mobility impairments to stay active.

^a^RPE: rating of perceived exertion.

^b^AIM: Acceptability of Intervention Measure.

^c^IAM: Intervention Appropriateness Measure.

^d^FIM: Feasibility of Intervention Measure.

## Discussion

### Principal Findings

This concurrent mixed methods pilot study evaluated a 6‑week AVG intervention delivered using the GAIMplank controller among adults with mobility impairments. The primary aims were to examine perceived exertion, enjoyment, and engagement during gameplay and to assess implementation outcomes—acceptability, appropriateness, and feasibility—using both quantitative ratings and qualitative feedback. These findings extend prior exergaming research by demonstrating the feasibility of a multisession intervention using an adapted controller specifically designed for seated users with mobility impairments, addressing an important accessibility gap identified in prior work. Overall, participants who completed the intervention described AVG gameplay as physically effortful without “feeling like exercise,” reported high enjoyment and engagement, and viewed the intervention as acceptable and suitable for their needs. In this small sample (n=6 enrolled; n=4 completers), median RPE scores of approximately 6 and high enjoyment and engagement scores (≥9) were observed across sessions (Table 4), supporting these overall perceptions. Qualitative findings generally aligned with quantitative results and highlighted practical implementation considerations, particularly intermittent controller responsiveness, that are important for future work.

### Comparison to Prior Work

Participants’ reported experiences suggest that AVG gameplay using the GAIMplank can elicit meaningful physical effort for seated users with mobility impairments, with perceived exertion often described as not feeling like exercise because participants were focused on the game. This perception aligns with evidence that perceived exertion during AVG can be lower than actual exertion when attention is focused on gameplay [20], while potentially minimizing the subjective burden often associated with traditional exercise. Several participants described exertion emerging as a byproduct of gameplay competition and strategy rather than conscious exercise effort. This is consistent with the observed median RPE values in the moderate range across most participants and sessions (Table 4). This phenomenon aligns with prior research, showing that perceived exertion during active gaming can differ from physiological demand, leading users to underestimate activity intensity when attention is directed toward gameplay rather than bodily sensations [32]. Such an effect may be particularly relevant for individuals who report that exercise is tiring or feels like hard work, as exergaming may support motivation and sustained engagement over time [16,32]. Consistent with prior studies demonstrating light‑to‑moderate intensity activity during AVG play [51-53], these findings support the feasibility of adapted exergaming as one option to increase movement opportunities for adults with mobility impairments.

Enjoyment and engagement are commonly identified as important factors for continued participation in exergaming and have been linked to energy expenditure during AVG play [8,31]. In this study, participants frequently described an initial learning curve and frustration, often associated with controller responsiveness or unfamiliarity with the control scheme, followed by increased enjoyment as they gained mastery and confidence. Median enjoyment and engagement scores were consistently high (typically ≥9; Tables 4 and 5), which was reflected in participants’ qualitative feedback. Competitive elements, clear objectives, and opportunities for progression appeared to support sustained engagement, suggesting that game selection and alignment with user preferences are critical components of intervention success. Importantly, participants remained engaged despite occasional technical challenges, which may reflect the value of gradual progression and consistent staff support. These findings are consistent with prior work in adults with physical disabilities, indicating that adapted exergaming systems can be both enjoyable and engaging when aligned with individual capabilities and preferences [8,37].

Implementation outcomes (AIM/IAM/FIM) and participant feedback suggested that the intervention was acceptable, appropriate, and feasible within a supervised laboratory environment [50]. Participants highlighted several factors that supported feasibility, including having multiple games to choose from, gradual increases in gameplay duration, and real‑time assistance from research staff. High ratings on the AIM, IAM, and FIM measures (Table 7) further support these perceptions of acceptability and feasibility. These findings align with recent adaptive gaming literature, emphasizing that successful implementation depends not only on device design but also on access to appropriate setup support, configuration, and user guidance [38]. At the same time, recurring controller unresponsiveness and delayed input occasionally disrupted gameplay and reduced enjoyment. For future research and translation, priorities include improving controller reliability and calibration, selecting games that are well‑matched to the adapted interface, and implementing structured onboarding procedures to promote early success and minimize frustration, as reflected in participant feedback and documented technical issues. Addressing these factors will be critical for translating adapted exergaming interventions beyond supervised settings into home and community settings.

Although standardized cognitive or mental health outcomes were not included in this study, participants frequently described mental stimulation, enhanced focus, and positive emotional experiences during gameplay. These observations are consistent with prior systematic review evidence, suggesting that exergaming may promote positive emotional experiences [27]. More recent evidence also suggests potential benefits for mood, social engagement, and certain cognitive outcomes in older adults, although findings remain heterogeneous across populations and outcome measures [28,29]. Given the exploratory nature of these observations and the absence of validated outcome measures in this study, conclusions regarding cognitive or psychological effects remain limited. Future studies should incorporate standardized assessments of mood, affect, and quality of life to more rigorously evaluate these potential benefits.

### Strengths and Limitations

This study had several limitations. The sample size was very small, with only 4 participants completing the full intervention; therefore, findings are preliminary, not generalizable, and limited to descriptive interpretation consistent with feasibility study aims. Two participants withdrew due to health reasons, which highlights the need for careful monitoring and support for individuals with complex health conditions. As a feasibility pilot, the study was designed primarily to evaluate usability, acceptability, and procedural viability rather than to estimate intervention effects. Therefore, quantitative outcomes should be interpreted descriptively and with caution. Technical issues with the GAIMplank interfered with some gameplay sessions (<20%) and may have confounded the measures of engagement and enjoyment. Exercise intensity was measured as perceived exertion, not objective physiological intensity. Finally, the absence of standardized cognitive or mental health measures limits conclusions regarding psychological outcomes, despite participant comments suggesting mental engagement and emotional benefits.

Despite these limitations, this pilot study contributes preliminary evidence supporting the feasibility and acceptability of adapted AVG for adults with mobility impairments who are unable to use commercial standing‑based systems [37,40,42]. The findings identify key design and implementation considerations—such as hardware reliability, game selection, progression, and support—that are likely to influence user experience and participation. More broadly, accessible seated exergaming has the potential to expand opportunities for enjoyable physical activity among individuals with mobility impairments [37,40,42]. As noted in a recent scoping review that evaluated the effects of assistive technology for adapted gaming, evidence of positive outcomes has been reported, but continued development of accessible gaming technologies is needed to support broader implementation [54]. Taken together, these findings illustrate how quantitative outcomes (Tables 4-7) and qualitative data converge to support the feasibility and acceptability of the intervention in this sample, as reflected in the integrated findings presented in Table 8. This study contributes to this growing area by evaluating not just usability, but the feasibility of repeated-session engagement using a seated, trunk-controlled interface.

### Future Directions

Future research should prioritize larger, controlled studies with improved hardware reliability, objective measures of physical activity and function, and validated psychosocial outcomes to evaluate effectiveness, longer‑term engagement, and scalability across settings.

### Conclusions

Findings from this mixed methods pilot study suggest that AVGs, delivered using an adapted controller like the GAIMplank, are a feasible and enjoyable way to promote physical activity among adults with mobility impairments. The study also points out practical issues that will matter for future use, including controller reliability, selecting games that work well with the controller, and having clear staff support and set-up procedures. More broadly, accessible seated exergaming may expand the options for enjoyable physical activity for people with mobility impairments. Future research should prioritize larger, controlled studies with improved hardware reliability and include objective measures of activity intensity and function, along with validated psychosocial measures. Longer-term studies are also needed to examine sustained use and potential benefits over time and to better understand what approaches best support continued participation.

## Data Availability

The datasets generated or analyzed during this study are available from the corresponding author on reasonable request.

## Abbreviations

AIM: Acceptability of Intervention Measure
AVG: active video game
EEQ: Exergame Enjoyment Questionnaire
FIM: Feasibility of Intervention Measure
IAM: Intervention Appropriateness Measure
MOEES: Multidimensional Outcome Exercise Expectations Scale
PACES: Physical Activity Enjoyment Scale
PASIPD: Physical Activity Scale for Individuals With Disabilities
RPE: rating of perceived exertion
